# 

**Published:** 1906-06

**Authors:** 


### BOOKS RECEIVED.

The Practice of Gynecology, in original contributions by American authors, edited by J. Wesley Bovee, M. D., Professor of Gynecology in George Washington University, Washington, D. C. In one octavo volume, containing 838 pages, with 382 engravings and 60 full page plates in colors and mono-chrome. Lea Brothers & Company, Philadelphia and New York. 1906. (Cloth, $6.00, leather, $7.00, half morocco, $8.00, net).

Transactions of the American Roentgen Ray Society. Sixth annual meeting held at Baltimore, September 28, 29 and 30, 1905. George C. Johnston, M.D., Secretary.

The Practical Medicine Series of Year Books. Ten volumes. Issued under the general editorial charge of Gustavus P. Head, M.D., Professor of Laryngology and Rhinology in the Chicago Post-Graduate Medical School. Vol. I. General Medicine, edited by Frank Billings and J. H. Salisbury. (Price, $1.25). Vol. II. General Surgery, edited by John B. Murphy. (Price, $2.00; entire series, $10.00). Chicago: The Year Book Publishers. 1906.

Human Sexuality. A Medico-Literary Treatise on the Laws. Anomalies, and Relations of Sex, with special reference to contrary sexual desire. By J. Richardson Parke, S.B., Ph.G., M.D. (Late Acting Assistant Surgeon, U. S. Army). Octavo, 476 pages. Professional Publishing Company, Philadelphia. 1906. (Price, $3.00 net).

